# Development of an EN8 Steel Stepped Rotor by a Novel Engraving Milling Technique

**DOI:** 10.3390/ma17071588

**Published:** 2024-03-30

**Authors:** Sujeet Kumar Chaubey, Kapil Gupta

**Affiliations:** Department of Mechanical and Industrial Engineering Technology, University of Johannesburg, Doornfontein Campus, Johannesburg 2028, South Africa; schaubey@uj.ac.za

**Keywords:** stepped rotor, engraving milling, EN8 steel, BBD-RSM, average roughness, machining time

## Abstract

The rotor or impeller is a rotational and key part of a pump and compressor. This article presents the detailed development process of a rotor of small size constructed from an EN8 steel cylindrical blank using a novel technique based on a computer numerical control engraving milling machine (CNC-EMM) equipped with a 4 mm tungsten carbide end mill cutter. We fabricated a total of twenty-eight stepped rotors following the Box–Behnken Design (BBD) DoE technique at fourteen distinct combinations of CNC-EMM variable parameters, namely rotational speed, feed, and plunge feed. Average roughness ‘*R_a_*’, an important surface quality indicator, has been considered and presented in this article, as a quality measure for the fabricated rotors. Feed and plunge feed have been identified as the most influencing variable parameters as per an analysis of variance (ANOVA) test. The lowest average roughness value obtained by this process for the rotor blade was 0.11 µm. A micrograph obtained from a field-emission scanning electron microscope (FE-SEM) showed a uniform and accurate tooth profile along with burr formation at corner edges. This study claims to establish engraving milling as a viable alternative to other manufacturing processes used for rotor blades. The findings of this study are useful to scholars, engineers, and researchers who are exploring new ways to fabricate mechanical parts and components.

## 1. Introduction

A rotor is an integral part of pumps, compressors, and turbines. It is a rotating component consisting of a series of several curved blades. In the case of a pump, its main function is to accelerate and increase the pressure and flow of fluid. Meanwhile, in a turbine, it converts the energy of the liquid into rotational motion [1,2]. The performance and durability of the rotor mainly depend on the surface finish and geometrical profile of the blades. The methods used to manufacture the rotors include casting; deposition techniques; Lithographie, Galanoformung, and Abformung (LIGA); and electric discharge machining (EDM) [3,4]. However, these manufacturing methods have certain restrictions and are not appropriate for producing rotors in large quantities. Furthermore, these methods are suitable for specific materials, such as casting and deposition methods for high-fluidity materials, LIGA for non-reflective materials, and EDM for electrically conductive materials [5].

Keeping the above challenges in mind, we attempted to explore a novel hybrid technique of engraving and milling for rotor fabrication. Engraving is a subtractive-type material removal process in which a rotating cutter is used to gradually remove a small amount of material from the surface of the part along a definite path in order to create the desired shape. It employs a set of rotary cutting tools to remove materials from the workpiece to form 3D effects. In this process, the workpiece is held in between the fixtures, and the cutting tool and worktable move to obtain the required engraving. CNC engraving is the modern version of engraving whereby automatic CNC technology guides the engraving tool with high accuracy and precision control to engrave letters or patterns into a surface. A wide range of 2D and 3D shapes and patterns can be engraved using the CNC engraving machining process. CNC milling engraving is used when the workpiece materials to engrave are composed of stainless steel, mild steel, aluminum, brass, wood, and plastics. Several industries have utilized CNC engraving for a wide variety of applications, such as sign-making, jewelry design, and industrial marking. It is also widely employed in tool and dies industries [6,7]. This hybrid method is not only fast and efficient, but also ecofriendly for the possible manufacturing of rotor type mechanical parts.

In the past, there have been some attempts to utilize laser engraving and develop a CNC engraving machine tool to engrave various materials [8,9,10,11,12]. With an aim to improve the performance of laser engraving machines by achieving precise motion at an appropriate speed, Martinov et al. [8] developed an algorithm and conducted an experimental study. They found a significant increase of up to 50% in engraving speed, simultaneously achieved with high product quality. Kumar et al. [9] developed an Arduino CNC-controlled, lightweight, portable, and inexpensive LEM for small- and medium-sized industries. The accuracy of laser engraving on UV-coated paper quality was investigated by Lei et al. [10]. They reported that better machining can be achieved with increased laser power and that the best precision can be obtained at a preset width of 0.26 mm and laser power of 11. By adding a work head replacement mechanism to a 3D printing machine, W. Durna et al. [11] developed a modified 3D printing machine that can work as a multifunctional CNC laser engraving machine. A low-cost LEM for 3D modeling and simulation was developed by Khalid et al. [12] using CREO 2.0 software. It was found that the parts manufactured by the laser engraving machine were of adequate quality.

The CNC engraving milling (CNC-EMM) process has also been used by some researchers for various purposes [13,14,15,16,17]. An open-architecture numerical control of motion and PLC for CNC-EMM was proposed by Lian et al. [13] to achieve high performance at high speed with greater precision. In another important work, a design strategy was made to develop a three-dimensional open-system-based CNC engraving milling machine for manufacturing parts from metallic and non-metallic materials [14]. The experiments showed that the developed machine could produce parts of superior quality with a high degree of efficiency. A useful study reported the development of a CNC engraving milling machine (CNC-EMM) to manufacture the printed circuit board (PCB) at a reasonable cost [15]. Bangse et al. [16] also attempted to design and develop a CNC router engraving machine with an accuracy of 99.5% along the X and Y axes and 96% along the Z axis. Similarly, Kumar et al. [17] designed and developed an Arduino-controlled CNC-EMM. It was claimed that the developed CNC-EMM could be used to mill, cut, and engrave a specific-sized workpiece. A recent study also worth mentioning is the one in which Brenci and Gurau [18] attempted to obtain good surface quality on beech wood using the profile milling technique. They identified feed speed as the most influential factor.

But the fabrication of rotors was only being performed by, EDM and casting technologies. Vdovin and Smelov [3] attempted to use sophisticated investment casting techniques and rapid prototyping technology to fabricate a miniature gas turbine engine (GTE) rotor. In another important work, Geng et al. [4] manufactured micro-sized GTE rotors by micro-EDM and micro-extrusion processes.

It was identified from the review of past work that rotor manufacturing is still a challenging task and that attempts to fabricate rotors by engraving milling are extremely limited. Considering this, the present work aims to explore and establish engraving milling as an alternative to traditional processes for rotor manufacturing. The scope of the work reported in this article includes the following: exploring the capability of CNC-EMM to manufacture the semi-open type stepped rotor from EN8 steel cylindrical blank; illustrating the complete sequence and steps of the engraving milling procedure of manufacturing the semi-open type stepped rotor by CNC-EMM; identifying the effect of engraving milling parameters namely rotational speed, feed, plunge feed on average roughness ‘*R_a_*’ to understand the fabrication; and micrograph analysis of the CNC-EMM-manufactured, semi-open-type stepped rotor to evaluate its surface quality.

## 2. Fabrication and Experimental Details

### 2.1. Materials and Methods

**Figure 1:**
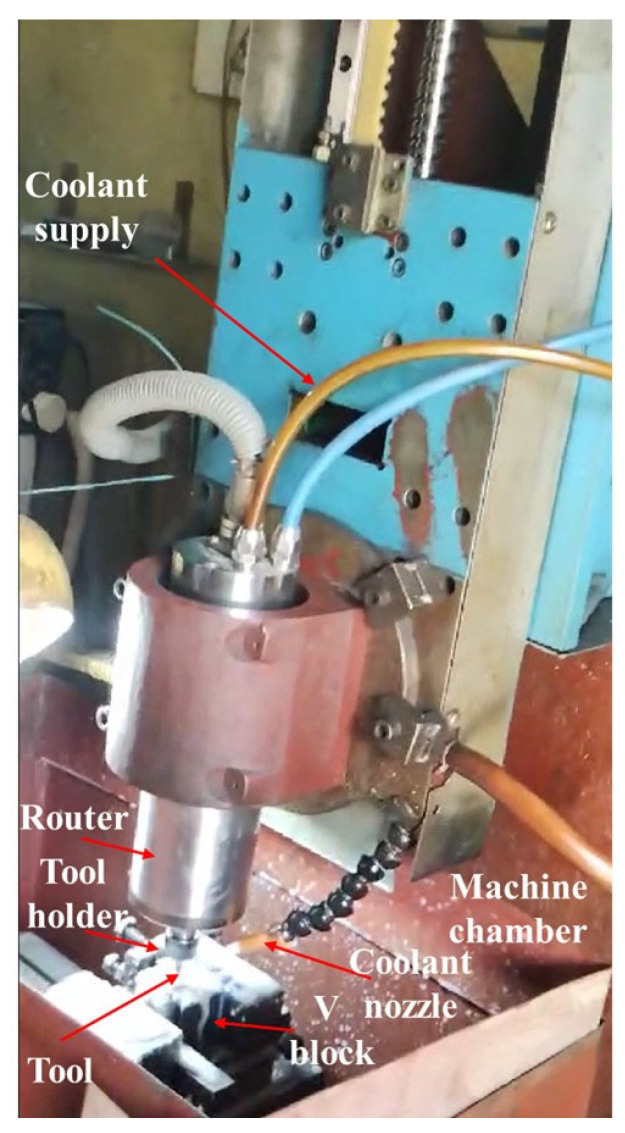
CNC engraving milling machine tool used in the present work.

**Figure 2:**
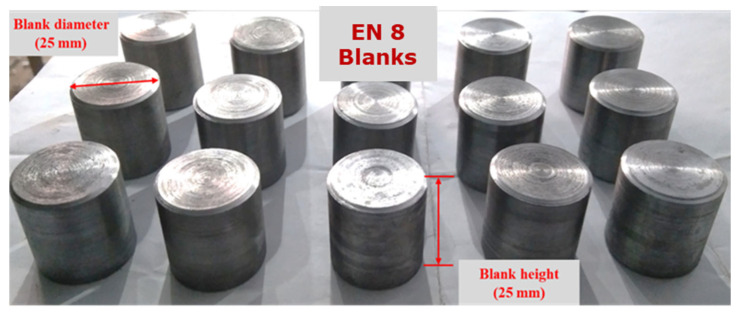
The EN8 steel cylindrical blanks used to fabricate rotors.

We have used an indigenously developed CNC engraving milling machine tool (CNC-EMM) setup as shown in Figure 1, to manufacture the small-sized, semi-open-type stepped rotors from EN8 steel blanks. The worktable can move in the X and Y directions. Meanwhile, the cutting tool can move up and down in the Z direction. The cutting tool held firmly in a CNC router rotates at high speed in clockwise and counterclockwise directions according to the part program. The end mill tungsten carbide tool (Fire-VHM Schaftra, Series: DIN 6527l R-N HRC 56; manufacture and country origin: Gurhing, Germany), having a 4 mm diameter and four flutes, was used to manufacture the stepped rotor from the EN8 steel cylindrical blank by CNC-EMM in the presence of continuous flowing coolant. The used end mill tool features four cutting edges and a right-hand helix. A very fine layer of titanium aluminum nitride (TiAlN) was applied to the tool’s surface. EN8 steel, also known as 080M40, is frequently utilized in automotive and general engineering applications because of its good machinability, toughness, and intermediate strength [19,20,21]. A few typical applications of EN8 carbon steel are as hydraulic fittings, machinery parts, gears, shafts, crankshafts, axles, spindles, fasteners, pins, studs, bolts, keys, and other parts. EN8 steel blanks having a diameter of 25 mm and height of 25 mm, as illustrated in Figure 2, were manufactured from a 450 mm long and 28 mm diameter EN8 stainless steel round bar using a manual lathe tool. The blank was then turned and drilled to make the stepped blank to manufacture the rotor with a hub, blind bore, and screw hole. The chemical composition (%) of the EN8 blank used in the present work is C: 0.36–0.44; Si: 0.1–0.4; Mn: 0.6–1.0; P: 0.05; S: 0.05; Cr: 0.3; Ni: 0.25; and the rest being Fe.

### 2.2. Procedure of Manufacturing the Small-Sized Stepped Rotor

In the engraving process, the cutting tool removes the material from the workpiece layer-by-layer in several steps to bring it to the desired shape and size. The cutting tool moves upward after each step of cutting, returns to its initial position, and then moves downward at a certain depth known as plunge down, as given in the part program. The values of 50 µm, 100 µm, and 100 µm are the values of the plunge down in stage 1, 2, and 3, respectively. In this study, each blade was manufactured using one hundred steps of the cutting tool in the first stage of engraving. Rotational speed indicates the rotation of the cutting tool in the clockwise direction. Feed indicates the movement of the workpiece in the X and Y directions to the cutting tool according to the part program. Therefore, higher values of feed result in a faster removal of materials from the workpiece in the predefined path according to the part program. The plunge feed indicates the speed at which the cutting tool moves to its initial position, including the plunge up and down after each step of cutting to perform the next step of cutting.

**Figure 3:**
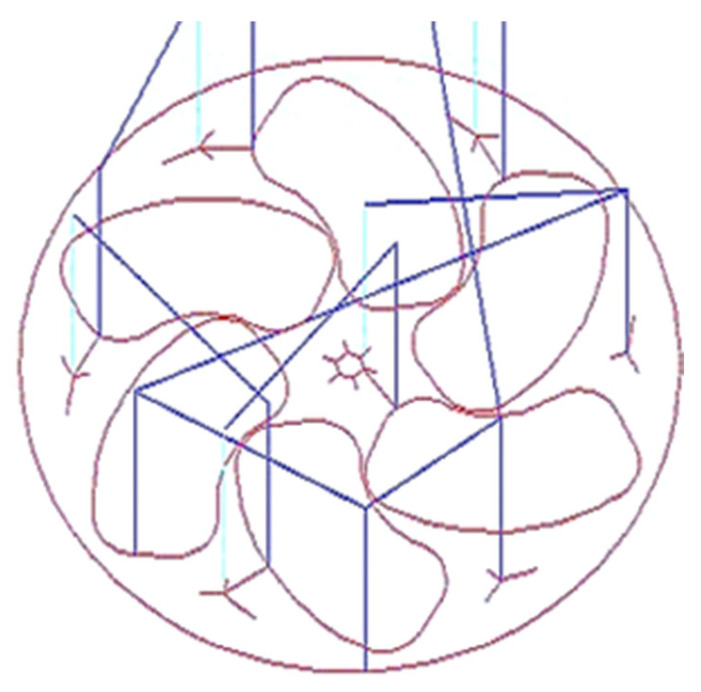
The 2D layout of the semi-open rotor. Blue line represents movement of tool and light marron colour represents the layout of blades and rotor circumference.

**Figure 4:**
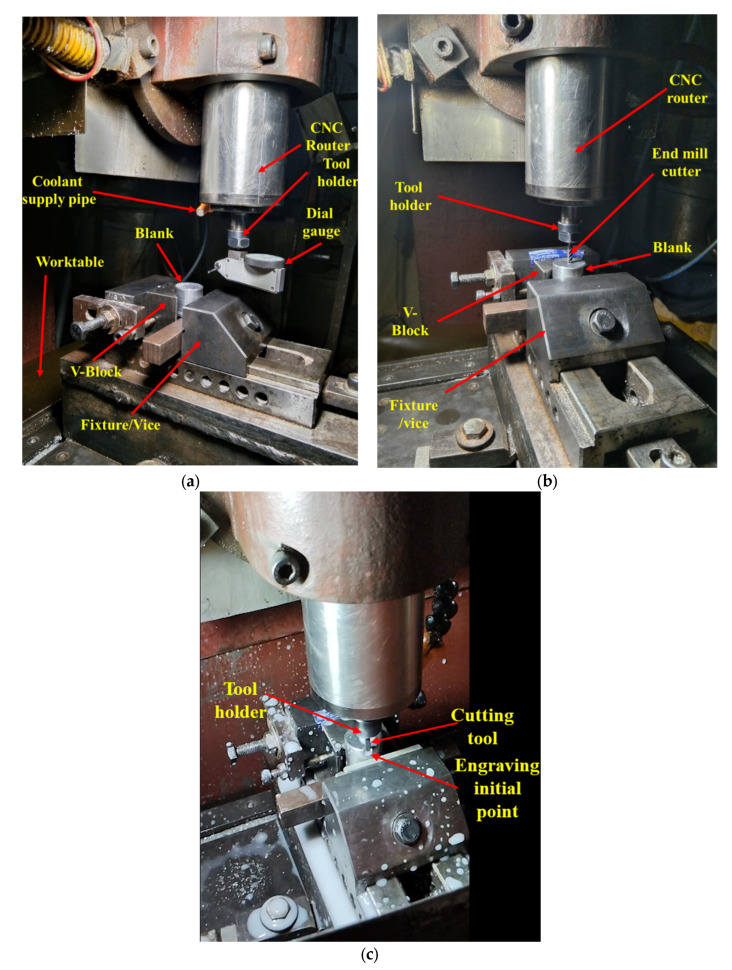
Different steps involved during the preparation phase of engraving by CNC-EMM: (**a**) measurement of the uniform flatness of the top surface of the blank with the help of a dial gauge; (**b**) Z height setting; (**c**) initial point of the machining phase.

**Figure 5:**
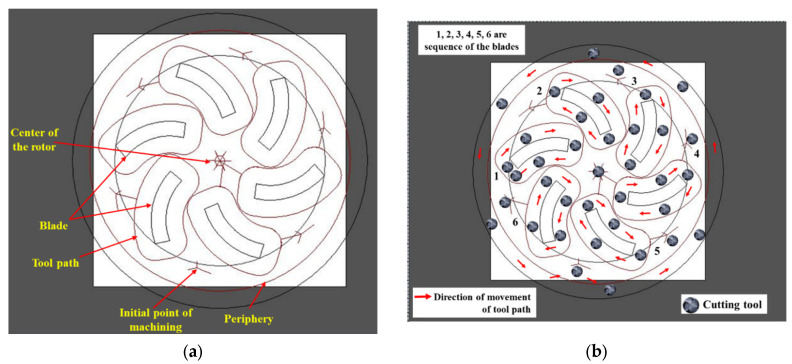
Layout of the rotor blade and cutting tool path: (**a**) Two-dimensional layout of the rotor and cutting tool; (**b**) direction of the movement of the tool path.

**Figure 6:**
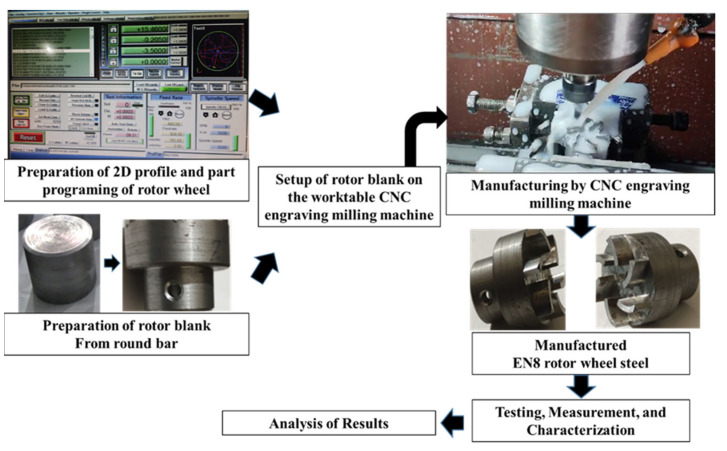
Overall process sequence for manufacturing rotor blades using CNC engraving milling.

Several steps are involved in manufacturing a semi-open-type stepped rotor using a CNC engraving milling machine equipped with a 4 mm diameter tungsten carbide end mill cutter. The sequence of machining steps involved in manufacturing the stepped rotor are as follows:

- Preparation of the blanks with a 25 mm diameter from the EN8 steel round bar using a manual lathe machine tool.
- The next step is the preparation of the stepped blank with a hub, bind bore, and screw hole from the blank by turning and drilling machining operations.
- After the preparation of the stepped blank, the next step is the preparation of a two-dimensional layout (Figure 3) of the semi-open-type stepped rotor with the help of *AutoCAD 2023*.
- *The next step is* the conversion of the *AutoCAD-prepared* two-dimensional geometrical layout of the rotor into a G-code and M-code part program.
- The next step is transferring the prepared part program to the CNC engraving milling machine with the help of a disk or pen drive.
- The next step is the referencing of the CNC engraving milling machine. CNC referencing is compulsory before starting the machining process in each experimental run.
- After that, the next step is clamping the designed fixture and V block on the worktable of the CNC engraving milling machine to firmly hold the stepped blank using screws.
- The next step is ensuring the firm and appropriate positioning of the prepared stepped blank in the V block with the help of the dial gauge, as shown in Figure 4a. Minor variations in the positioning of the blank and flatness of the top surface significantly increase the noise and tool wear and reduce the dimensional accuracy as well as the surface quality of the manufactured turbine.
- The next step is the setting of the Z height of the cutting tool just above the top face of the blank with the help of a special gauge for safety purposes, as shown in Figure 4b. It prevents the cutting tool from coming in direct contact with the blank for machining. The movement of the cutting tool will be stopped at this height before starting the machining to avoid any risk of damage due to program or operator error.
- The window-based Mac 3 software is incorporated with the used CNC-EMM approach to read the part program and direct the cutting tool movement on the desired path of the part program.
- Machining initiates with cutting and preparing the exact periphery as per the part program shown in Figure 4c. Cutting tools rotate in a clockwise direction.
- The small-sized stepped rotor is manufactured into three machining stages, known as the rough cut (RC), semi-finished cut (SFC), and finished cut (FC). A different part program was prepared for each stage.
- The cutting tool rotates and moves in a clockwise direction during the engraving blade. The layout of the rotor blade and the direction of the tool path are shown in Figure 5. In each movement, the tool removes 50 µm of material in downward Z directions. Thus, the tool repeats the same path a hundred times to engrave a single blade with 5 mm height.
- Figure 6 shows the whole process sequence and engraving of the rotor blades by CNC-EMM.
- After cutting the steeped rotor, the next step is to perform measurements of the average surface roughness and capture micrographs of the manufactured rotor blade using a field-emission scanning electron microscope (FE-SEM).

### 2.3. Experimentation and Measurements

The Box–Behnken design (BBD) technique of response surface methodology (RSM) is one of the most extensively used and prominent techniques for designing experiments in various engineering fields and applications, including manufacturing [22,23]. In this work, BBD was employed to conduct experiments during CNC-EMM for manufacturing the steeped rotors. The fourteen different combinations of CNC-EMM variable parameters were designed to conduct the experiments. Each combination was repeated twice; thus, a total of twenty-eight rotors have been manufactured in this study. for every individual experiment, a new end mill has been used. Rotational speed ‘*R_S_*’ (5500–6000–6500 rpm), feed ‘*f*’ (800–900–1000 mm/min), and plunge feed ‘*P_f_*’ (100–125–150 mm/min) were varied during experimentation to identify their effect on average roughness ‘*R_a_*’. Blank size, blank and cutting tool materials, coolant, and plunge down were the constant parameters.

The average roughness *R_a_* is an important parameter for analyzing the wear behavior and tribological characteristics of any surface [24,25]. In manufacturing, *R_a_* is used as a quality indicator for the fabricated surfaces. In this work as well, *R_a_* has been considered as an important machinability indicator. The *R_a_* of the machined surface of rotor blades was measured using the roughness tester of Mahr Metrology, Göttingen, Germany. For every single rotor, average roughness was measured at two different locations on the two different blades. The direction of measurement was opposite to the direction of end mill travel. An average value of the two replications for each experiment has been considered. To capture the micrographs of the blades of rotors, A field-emission scanning electron microscope ‘FE-SEM’ of Carl Zeiss, Jena, Germany, was also used. Figure 6 depicts the overall process steps adopted to manufacture rotor wheels in this work.

## 3. Results and Discussion

Experimentation results are discussed in this section. Table 1 presents all fourteen combinations of engraving milling parameters and the corresponding values of *R_a_* which are the average of two replications. An analysis of variance (ANOVA) test is the most important statistical tool for measuring the fitness of the data and model adequacy as well as identifying the influential parameters [26,27]. The ANOVA results for this study are given in Table 2. The ANOVA test was carried out using a 95% confidence interval of *p*-values (i.e., *p*-values must be less than 0.05), which is also discussed in this section. It has been observed that experiment 14 produced a maximum *R_a_* value (0.55 µm), and a minimum *R_a_* value (0.11 µm) was obtained in experiment 1.

**Figure 7:**
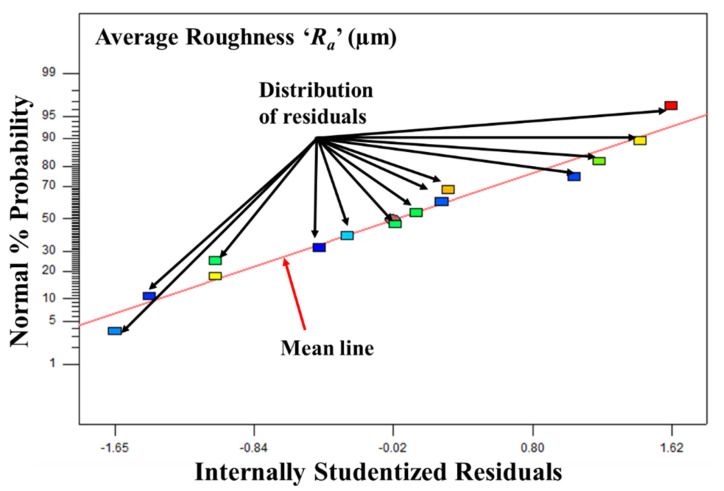
Plot of residual analysis for *Ra*.

The following can be concluded from the ANOVA study:

- The developed linear model of average roughness ‘*R_a_*’ is significant as their ‘Prob > F’ (i.e., *p* values) is less than 0.05.
- Values of ‘Prob > F’ that are less than 0.05 indicate that the respective model terms are significant. Therefore, feed ‘*f*’ and plunge feed ‘*P_f_*’ were found to be statistically significant for average roughness ‘*R_a_*’.
- The F-value of 10.45 for average roughness ‘*R_a_*’ suggests that the model is significant.
- The lack of fit F-values are non-significant relative to the pure error for average roughness ‘*R_a_*’. The non-significant lack of fit is good and indicates that the developed model correctly fits the experimental data.
- Adequate precision indicates the signal-to-noise ratio, and its value being greater than four indicates an adequate signal. The value of adequate precision for *R_a_* is 10.08.
- The residuals of each experiment are concentrated around the mean line (Figure 7). Therefore, they exhibit a normal distribution.
- The developed empirical equation 1 is linear (i.e., without square terms or interaction terms) and can be used for the future prediction of the values of average roughness ‘*R_a_*’.

(1) $$R_{t}=-1.76554+8.75000E-005\;S_{R}+1.06250E-003\;f+4.60000E-003\;P_{f}$$

**Figure 8:**
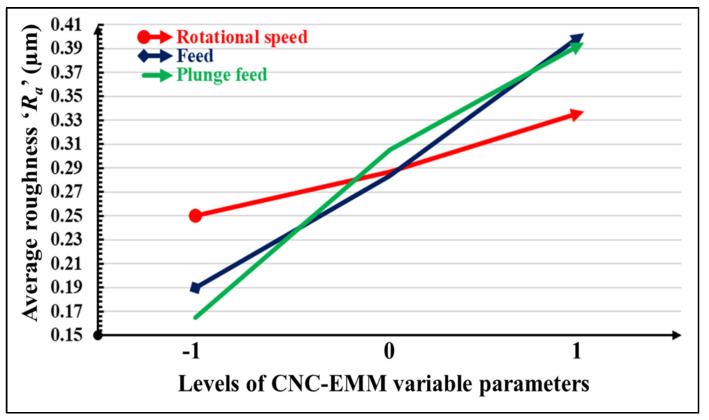
Variation in average roughness ‘*R_a_*’ with rotational speed, feed, and plunge feed.

Figure 8 depicts the variation in average roughness ‘*R_a_*’ with the rotational speed ‘*S_R_*’, feed ‘*f*’, and plunge feed *P_f_*’. Average roughness increases with the increase in all variable parameters. It can be observed that rotational speed has less impact on average roughness ‘*R_a_*’. Average roughness ‘*R_a_*’ is slightly increased (0.25–0.29 µm) with rotational speed. Meanwhile, feed and plunge feed both have a significant influence on average roughness ‘*R_a_*’. Average roughness ‘*R_a_*’ increases linearly with the increase in feed and plunge feed. The feed has more impact on the average roughness ‘*R_a_*’ compared with the plunge feed and rotational speed. These observations can be explained by the fact that a higher feed increases the movement of the workpiece according to the tool path. Plunge feed also reduces the time required to plunge up, return to its initial position, and plunge to a certain depth according to the part program for engraving the rotor blades. Therefore, higher values of feed and plunge feed reduce the overall time required to engrave the blade, resulting in a poor surface finish due to excessive heat generation, tool wear, and deposition of removed particles on the engraved surface in the absence of flushing.

**Figure 9:**
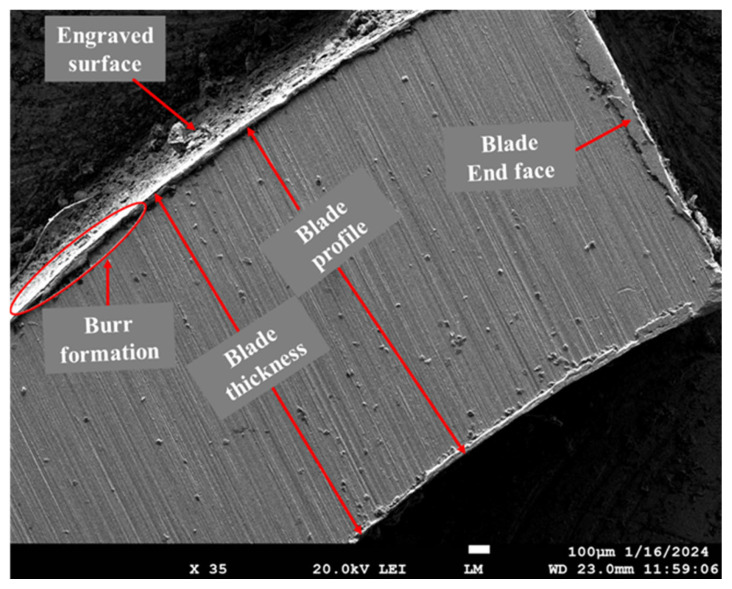
FE-SEM micrographs of the rotor blade with the lowest surface roughness, manufactured at a machining combination of 5500 rpm rotational speed, 800 mm/min feed, and 125 mm/min plunge feed.

The FE-SEM micrograph of the rotor that has the lowest average roughness value was also captured. According to Table 1 and Figure 8, experiment 1 (i.e., machining combination of 5500 rpm rotational speed, 800 mm/min feed, and 125 mm/min plunge feed) has the lowest average roughness. Figure 9 shows an FE-SEM micrograph of the blade of the best finish rotor, captured at a magnification of 35. It can be observed from the micrograph that the blade has a uniform profile with minute burr formation at the corner edges.

It is worth mentioning that the lowest average roughness (0.11 µm) obtained on the rotor wheel fabricated in the present work is much better than that reported in the past work (1.38 µm) [28]. Moreover, electric discharge machining, which was used in that previous work, is a slow process and not suitable for mass production.

## 4. Conclusions

This article has presented the detailed steps of the development of small-size stepped rotors from EN8 steel using an engraving milling process on a CNC machine tool using a 4 mm diameter end mill cutter. An experimentation study was also conducted and found a good surface quality, after measuring average roughness *R_a_*, of the fabricated rotor blades. Feed and plunge feed have a major impact on average roughness ‘*R_a_*’ compared with rotational speed. Average roughness ‘*R_a_*’ increased linearly with the increase in feed rate and plunge feed. The best surface finish, i.e., the least value of average roughness *R_a_*, is 0.11 µm for the stepped rotor blade manufactured at 5500 rpm rotational speed, 800 mm/min feed, and 125 mm/min plunge feed. The maximum value of the average roughness ‘*R_a_*’ (i.e., 0.55 µm) was observed at 6500 rpm rotational speed, 1000 mm/min feed, and 125 mm/min plunge feed. The SEM micrograph of the best finish stepped rotor showed a uniform and accurate geometrical profile with slight burr formation and the deposition of fine particles on the surface of the blade. The best surface roughness for the rotor blade achieved by CNC engraving milling is better than that obtained in the previous work based on rotor manufacturing by other processes. The development of rotors by CNC engraving milling is simple enough, with no complications related to typical programming and machine tools. Considering the outcomes and findings, it can be concluded that this study establishes the engraving milling process as an alternative to conventional processes of manufacturing quality rotors. This opens up new avenues of future research in typical mechanical parts and components manufacturing using the engraving milling technique. A possible future research scope includes the fabrication of rotors of other materials using the engraving milling technique, manufacturing of gears and other mechanical components by this technique, process optimization, life cycle engineering and analysis of engraving milling technique, etc.

## Figures and Tables

**Table 1:** Results of the CNC-EMM of rotor blades with experimental combinations and average surface roughness values.

Expt. No.	CNC-EMM Variable Parameters			Response/Performance Measure
	Rotational Speed ‘*S_R_*’ (rpm)	Feed ‘*f*’ (mm/min)	Plunge Feed ‘*P_f_*’ (mm/min)	Average Roughness ‘*R_a_*’ (µm)
1	5500	800	125	0.11
2	5500	900	150	0.29
3	5500	900	100	0.15
4	5500	1000	125	0.45
5	6000	800	150	0.38
6	6000	800	100	0.14
7	6000	900	125	0.3
8	6000	900	125	0.29
9	6000	1000	100	0.17
10	6000	1000	150	0.44
11	6500	800	125	0.13
12	6500	900	150	0.47
13	6500	900	100	0.2
14	6500	1000	125	0.55

**Table 2:** Results of the ANOVA test for average roughness ‘*R_a_*’.

Source	Sum of Squares	DF	Mean Squares	F-Value	*p*-Value (Prob > F)	Remarks
Model	0.21	3	0.070	10.45	0.0020	Significant
Rotating speed ‘*S_R_*’	0.015	1	0.015	2.27	0.1629	Not significant
Feed ‘*f*’	0.090	1	0.090	13.39	0.0044	Significant
Plunge feed ‘*P_f_*’	0.11	1	0.11	15.68	0.0027	Significant
Residual	0.067	10	6.747 × 10^−3^			
Lack of Fit	0.067	9	7.491 × 10^−3^	149.82	0.0633	Not significant
Pure Error	5.000 × 10^−5^	1	5.000 × 10^−5^			
Cor Total	0.28	13				

*R*-squared: 0.758; adequate precision: 10.08; press: 0.15.

## Data Availability

Data are contained within the article.

## References

[1] Ahmad F., Kumar P., Patil P.P., Dobriyal R., Avikal S. (2023). Comparative analysis of two and four blades Quadcopter propellers based on finite element method. Mater. Today Proc..

[2] Soori M., Asmael M. (2023). Minimization of deflection error in five axis milling of impeller blades. Facta Univ. Ser. Mech. Eng..

[3] Vdovin R.A., Smelov V.G. (2017). Design and optimization of the micro-engine turbine rotor manufacturing using the rapid pro-totyping technology. IOP Conf. Ser. Mater. Sci. Eng..

[4] Geng X., Chi G., Wang Y., Wang Z. (2014). High-efficiency Approach for Fabricating MTE Rotor by Micro-EDM and Mi-cro-extrusion. Chinese. J. Mech. Eng..

[5] Jain N.K., Chaubey S.K., Hashmi M.S.J. (2016). Review of Miniature Gear Manufacturing. Comprehensive Materials Finishing.

[6] Suharto S., Suryanto S., Sarana S., Purbono K. (2021). Application of CNC machine router 3-Axis for making of Engraved granite or marble. IOP Conf. Ser. Mater. Sci. Eng..

[7] Peng T.-Y., Shimoe S., Higo M., Kato M., Hirata I., Iwaguro S., Kaku M. (2024). Effect of laser engraving on shear bond strength of polyetheretherketone to indirect composite and denture-base resins. J. Dent. Sci..

[8] Martinov G.M., Obuhov A.I., Martinova L.I., Grigoriev A.S. (2016). An Approach to Building a Specialized CNC System for Laser Engraving Machining. Procedia CIRP.

[9] Kumar P.J., Tarun A.S.S., Gowtham M., Rao P.T., Yashwanth G. (2018). Design and fabrication of portable laser cutting and en-graving machine. Int. J. Eng. Technol..

[10] Lei X., Liu S., Wu N., Ge Y., Hou H., Liu P. (2020). Experimental investigation of laser engraving quality on paper. Appl. Opt..

[11] Durna A., Fries J., Hrabovsky L., Sliva A., Zarnovsky J. (2020). Research and Development of Laser Engraving and Material Cutting Machine from 3D Printer. Manag. Syst. Prod. Eng..

[12] Khalid M.S., Jaleed S.M., Zafar A., Khan S.A., Rehman H.Z.U., Khan Z.H. Design and Experimental Verification of a Laser Engraving Machine. Proceedings of the 2023 International Conference on Emerging Power Technologies (ICEPT).

[13] Lian P., Wen Y., Yu P., Ke L., Wei L., Hui X. Research of PMAC-based open NC system for Engraving and milling machine. Proceedings of the 2010 International Conference on Mechanic Automation and Control Engineering (MACE).

[14] Cao S.K., Sui Z.M., Liu L.N., Wang G.C., Song W.W. (2011). Engraving and Milling Machine Design Based on Open CNC System. Appl. Mech. Mater..

[15] Choudhary R., Titus S.D., Akshaya P., Mathew J.A., Balaji N. CNC PCB milling and wood engraving machine. Proceedings of the 2017 International Conference On Smart Technologies For Smart Nation (SmartTechCon).

[16] Bangse K., Wibolo A., Wiryanta I.K.E.H. (2020). Design and fabrication of a CNC router machine for wood engraving. J. Phys. Conf. Ser..

[17] Kumar J., Singh S., Tripathi S., Shukla V., Pathak S. (2022). Design and fabrication of 3-axis CNC milling machine using additive manufacturing. Mater. Today Proc..

[18] Brenci L.-M., Gurău L. (2023). A Stratified Characterization of Surface Quality of Beech Processed by Profile Milling. Appl. Sci..

[19] Chandran R., Udhayaraj S., Eazhil K.M. (2022). Effect of the heat-treatment process on the mechanical and microstructure properties of EN8 steel. Int. J. Surf. Engg. Interdiscip. Mater. Sci..

[20] Schlegel J. (2023). The World of Steel.

[21] Bolton W., Higgins R.A. (2020). Materials for Engineers and Technicians.

[22] Montgomery D.C. (2019). Design and Analysis of Experiments.

[23] Lorenzen T.J., Anderson V.L. (2019). Design of Experiments.

[24] Davim J.P. (2010). Surface Integrity in Machining.

[25] Bhushan B. (2013). Principles and Applications of Tribology.

[26] Moodie P.F., Johnson D.E. (2022). Applied Regression and ANOVA Using SAS.

[27] Herzog M.H., Francis G., Clarke A., ANOVA (2019). Understanding Statistics and Experimental Design.

[28] Sankar S., Jessin T.A., Naik M.V. (2016). Modeling of EDM electrodes for development of LPOT turbine rotor and optimization of parameters for attenuate portioned electrode by Taguchi based Grey Relational Analysis. Int. J. Adv. Eng. Manag. Sci..

